# Finite element analysis of the initial stability of arthroscopic ankle arthrodesis with three-screw fixation: posteromedial versus posterolateral home-run screw

**DOI:** 10.1186/s13018-020-01767-7

**Published:** 2020-07-10

**Authors:** Sen Wang, Jian Yu, Xin Ma, Dahang Zhao, Xiang Geng, Jiazhang Huang, Xu Wang

**Affiliations:** Department of Orthopedics, Huashan Hospital, Fudan University, 12 Middle Wulumuqi Road, Shanghai, 200040 China

**Keywords:** Finite element method, Screw configuration, Biomechanics, Arthroscopic ankle arthrodesis

## Abstract

**Objective:**

Arthroscopic ankle arthrodesis (AAA) is a standard surgical method for the treatment of advanced traumatic ankle arthritis and has become more popular due to its advantages. To fix the tibiotalar joint, the use of three percutaneous screws is considered to have better mechanical stability than the use of two screws. However, it is sometimes difficult to insert three screws because they might block each other due to the small area of the tibiotalar joint surface and the large diameter of the screws; few articles illustrate how to insert three screws without the screws disturbing each other. The purpose of this study is to explore possible screw configurations of tripod fixation in arthroscopic ankle arthrodesis that avoid the collision of screws and yield better biomechanical performance.

**Methods:**

We used the finite element method to examine the impact of different screw positions and orientations on the biomechanical characteristics of a three-dimensional (3D) ankle model. Maximum and average micromotion, pressure on the articular surface, and von Mises stress values of the tibia and the talus were used to evaluate the initial stability of the ankle.

**Results:**

Five kinds of three-screw configurations were identified, and finite element analysis results suggested that configurations with the posteromedial home-run screw presented lower micromotion (maximum, 17.96 ± 7.49 μm versus 22.52 ± 12.8 μm; mean, 4.88 ± 1.89 μm versus 5.19 ± 1.92 μm) (especially configuration 3) and better screw distributions on the articular surface than those with the posterolateral home-run screw.

**Conclusion:**

Screw configurations with the posteromedial home-run screw avoid collision and are more biomechanically stable than those with the posterolateral home-run screw. Thus, inserting the home-run screw through the posteromedial approach is recommended for clinical practice.

## Background

Ankle arthrodesis (AA) is the standard surgical method for the treatment of advanced traumatic ankle arthritis [1]. It can relieve pain and help patients restore normal walking through the fusion of the tibiotalar joint. AA can be grouped into open surgery, arthroscopic ankle arthrodesis (AAA), and external fixation fusion. In recent years, AAA has become increasingly popular with foot and ankle surgeons due to its advantages, such as being minimally invasive, having fewer complications (especially skin complications), and its rapid recovery [2–4]. A recent systematic review found that AAA has higher clinical scores, fewer complications, shorter hospital stays, and less bleeding than open ankle fusion, while the overall fusion and reoperation rates of these methods are similar [5].

To perform an AAA, the standard ankle arthroscopy approach will be used, and articular cartilage and subchondral bone will be removed arthroscopically. Then, two or three cannulated compression screws will be inserted percutaneously to fix the tibiotalar joint [6]. There is currently no consensus in the literature as to whether two or three screws should be used [7, 8]. However, Alonso-Vazquez et al. [9] conducted a finite element analysis and found that the use of three screws has better mechanical stability than the use of two screws. In addition, Goetzmann et al. [10] retrospectively analyzed 111 AAA cases and found that the fusion rate of three screws was higher than that of two screws, and the time required for fusion was shorter with three screws.

However, it is sometimes difficult to insert three screws because they might block each other due to the small area of the tibiotalar joint surface and the large diameter of the screws (cannulated screws with a diameter of 6.5–7.5 mm are generally used during surgery [1, 11]) so that only two screws can be inserted. Few articles illustrate how to insert three screws without the screws disturbing each other. Schuberth et al. [12] introduced the tripod fixation technique, but their methods did not address the possibility of collision, and mechanical stability was not taken into account.

At present, the finite element method is widely used in the field of orthopedic biomechanics. Vazquez et al. [13] used finite element analysis to compare the effect of two joint surface processing methods on the initial stability of ankle fusion and found that better initial stability was reported when the joint contours were preserved rather than resected. Zhu et al. [14] compared the effects of three kinds of 2-screw configurations on the loading stress of the tibiotalar joint with a similar method.

Therefore, the purpose of this study is to explore possible configurations of tripod fixation in arthroscopic ankle arthrodesis that avoid the collision of screws and to compare biomechanical stability through the finite element method.

## Materials and methods

### Geometric characteristics of the finite element model

**Fig. 1:**
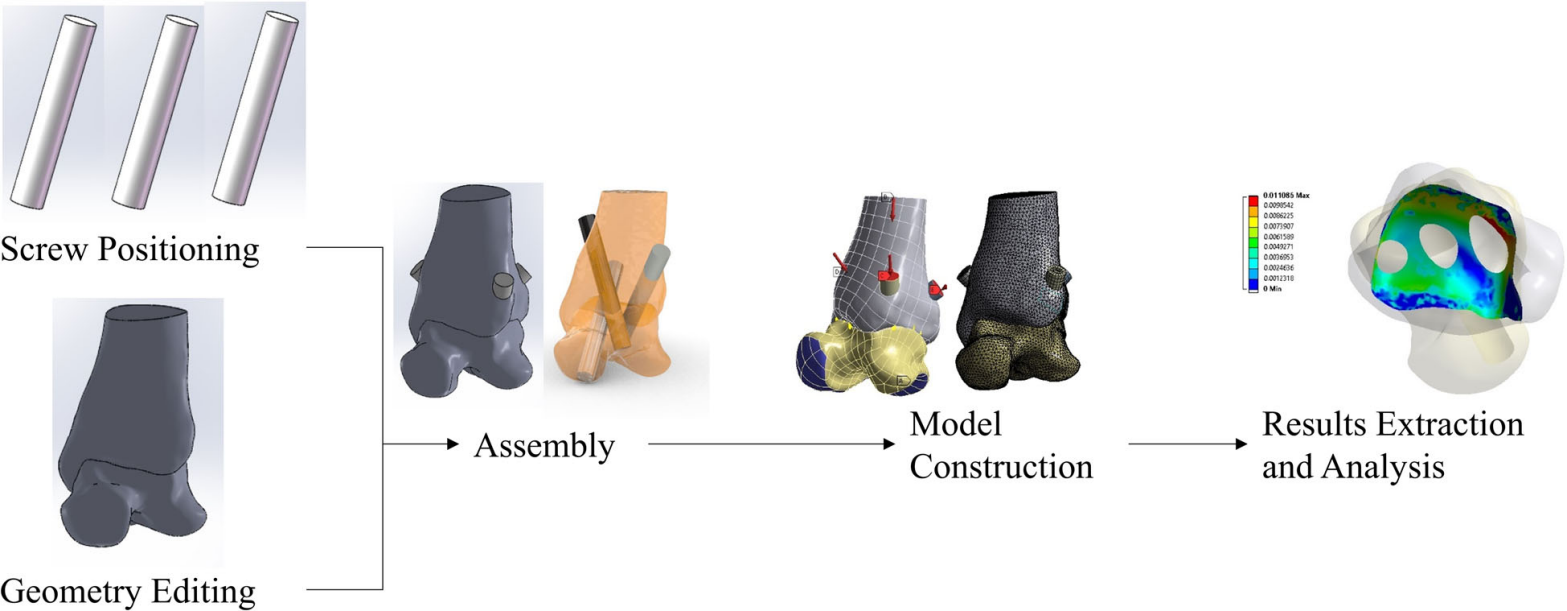
Schematic diagrams of the modeling and analysis procedure

A 24-year-old healthy male volunteer who was 175 cm tall and weighed 70 kg was enrolled. X-ray examination showed no foot or lower limb fracture, no tumors, and no deformities, and the patient had no history of surgery. A computed tomography scan was taken of the right foot and ankle by Siemens computed tomography (CT) system (Sensation 64, Siemens Healthcare, Germany) with a slice thickness of 0.6 mm in our hospital. The newly developed foot and ankle brace [15] was used to maintain a neutral position during scanning. A total of 261 2D tomographic images were collected and saved in DICOM format. The scanned computed tomography (CT) file was then imported into the medical 3D modeling software Mimics (Materialise, Leuven, Belgium), and the three-dimensional geometric point clouds of the tibia and the talus were obtained by threshold segmentation and manual segmentation. To reduce the computational complexity, the reverse engineering software Geomagic (Geomagic studio 10.0, Geomagic, Research Triangle Park, NC) was used to homogenize the point clouds and encapsulate them into a triangular mesh surface for softening, repairing, and removing spikes. The surface was converted into a NURBS surface and then imported into 3D CAD software SolidWorks (Dassault Systems, France) to form a solid model in which the talus was moved up 1.8 mm along the longitudinal axis of the tibia to fill the joint space, and the articular surface of the distal tibia was trimmed to match the trochlea of talus as the ankle arthrodesis required removal of the articular cartilage and a good fit between the tibia and the talus. Considering that the diameter of cancellous screws commonly used in ankle arthrodesis is generally less than 8 mm, in this study, the three cancellous bone screws were simplified into cylinders with a diameter of 8 mm and added to the tibia-talus fusion model (see Fig. 1 for a schematic diagram).

### Exploring possible screw configurations

The SolidWorks software was used by clinicians (XM, XW) with experience in ankle arthrodesis surgery to explore the possible configurations of three screws through which the following four goals could be achieved. First, there was no collision of screws, and the portion of screws within bone was as long as possible. The positions where screws passed through the articular surface were evenly distributed on the trochlea of the talus. Finally, no screws penetrate the contralateral bone cortex.

Ankle arthrodesis models of various screw configurations were then imported into the finite element analysis software ANSYS Workbench (ANSYS Inc., Canonsburg, PA, USA) for material property assignment, meshing, interaction relationship definition, and boundary condition setting and calculation.

### Material properties, meshing, and interaction relationship definition of the finite element model

The tissues were set as an isotropic linear elastic material, and the material parameters for bone and screw were assigned in accordance with the literature [14, 16]. The Young’s modulus of the tibia and the talus was defined as 837 MPa and 13,000 MPa, respectively, and Poisson’s ratio was 0.3 [16]. Screws were regarded as incompressible material, Young’s modulus was defined as 110,000 MPa, and Poisson’s ratio was 0.4. Bones were divided by quadratic four-node tetrahedron elements, and cylinder screws were divided by hexahedron elements. A 1.5-mm mesh size was used determined by a mesh convergence test. The interaction of bones and screws is listed in Table 1.

**Table 1:** Interaction definition [14]

Contact	Interaction type
Tibia and talus	Frictional, coefficient of friction is 0.1
Screws and tibia	Tie
Screws and talus	Frictionless

### Loading and boundary settings

The mid-stance phase was simulated in these models. Vertical loads that were half weight were applied to the upper tibia surface. The plantar surface of the talus was completely fixed. Meanwhile, the effect of external force on primary stability was evaluated for early postoperative patients with plaster [13]. We applied a uniform pressure distribution of 50 MPa perpendicular to the top surface of each screw to simulate the external force [14]. Additionally, 10 Nm torque was applied in different directions to the upper tibia surface to simulate the load of dorsiflexion, internal rotation, and external rotation [9] (Fig. 1).

In this study, a finite element model based on 3D reconstruction of CT scan images was used to simulate biomechanics after ankle arthrodesis. The maximum and mean von Mises stress values at both the tibia and the talus were calculated to evaluate the stress distribution and stress transition to the screws. The maximum and mean micromotions at the fused articular surface were analyzed to evaluate the primary stability following ankle arthrodesis. We simulated four common clinical ankle stress scenarios (standing weight-bearing, dorsiflexion, internal rotation, and external rotation) and calculated the maximum and average von Mises stress values at bones and the maximum and average micromotion of the articular surface.

## Results

### Screw configurations

**Fig. 2:**
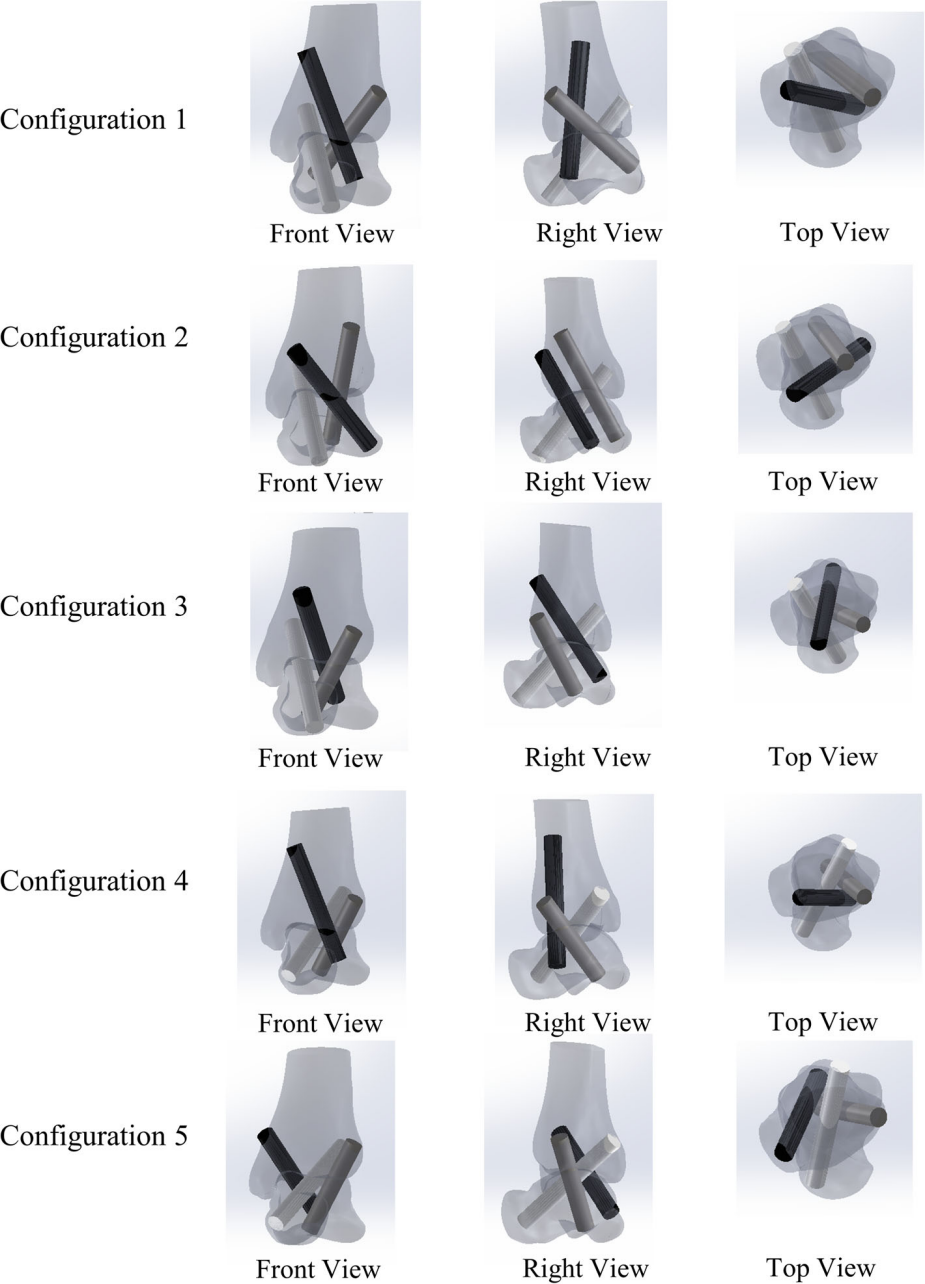
Different views of 5 screws configurations. (Images are front, left, and top views of each configuration, respectively.) (The home-run screw is labeled “S1” in white. The anterolateral screw is labeled “S2” in gray. The medial screw is labeled “S3” in black.)

Five possible screw configurations were identified (Fig. 2 and Table 2). Configurations 1–3 contain the posteromedial home-run screw, while configurations 4 and 5 contain the posterolateral home-run screw. For configuration 2, the anterolateral screw points to the posterolateral corner of the talus and provides more space for S3 insertion, which is different from that of configuration 1. For configuration 3, the anterolateral screw (S2) runs below the medial screw (S3), unlike configurations 1 and 2.

**Table 2:** A list of details of five possible screw configurations

Screw configurations	From	To	Length inside the bone (mm)
1 Posteromedial home-run	S1 posteromedial	Talar head-neck junction	56.1
	S2 anterolateral	Posteromedial corner of talus	48.6
	S3 medial	Lateral process of talus	55.8
	Total length		160.5
2 Posteromedial home-run	S1 posteromedial	Talar head-neck junction	56
	S2 anterolateral	Posterolateral corner of talus	60
	S3 anteromedial	Lateral process of talus	57.3
	Total length		173.3
3 Posteromedial home-run	S1 posteromedial	Talar head-neck junction	55.9
	S2 anterolateral	Posteromedial corner of talus	37.7
	S3 anteromedial	Posterolateral corner of talus	56.8
	Total length		150.4
4 Posterolateral home-run	S1 posterolateral	Talar head-neck junction	58.8
	S2 anterolateral	Posteromedial corner of talus	44.8
	S3 medial	Lateral process of talus	59.7
	Total length		163.3
5 Posterolateral home-run	S1 posterolateral	Talar head-neck junction	58
	S2 anterolateral	Posteromedial corner of talus	45.4
	S3 medial	Posterolateral corner of talus	47.4
	Total length		150.8

### Biomechanical analysis

Our results showed that all three-screw layouts had a lower contact pressure and micromotion on the articular surface than two-screw configurations [13, 14]. For all layouts, the maximum von Mises stress values at the tibia and the talus were 23.36 MPa (standard deviation (SD) 6.06 MPa) and 45.81 MPa (SD 8.15 MPa), respectively. Additionally, the mean von Mises stress values at the tibia and the talus were 2.10 MPa (SD 0.39 MPa) and 4.10 MPa (SD 0.61 MPa), respectively. At fusion site, the maximum contact pressure was 25.82 MPa (SD 5.23 MPa), and the mean contact pressure was 2.79 MPa (SD 0.27 MPa). The maximum micromotion on the articular surface was 19.79 μm (SD 10.21 μm), and the mean micromotion on the articular surface was 5.00 μm (SD 1.91 μm).

**Fig. 3:**
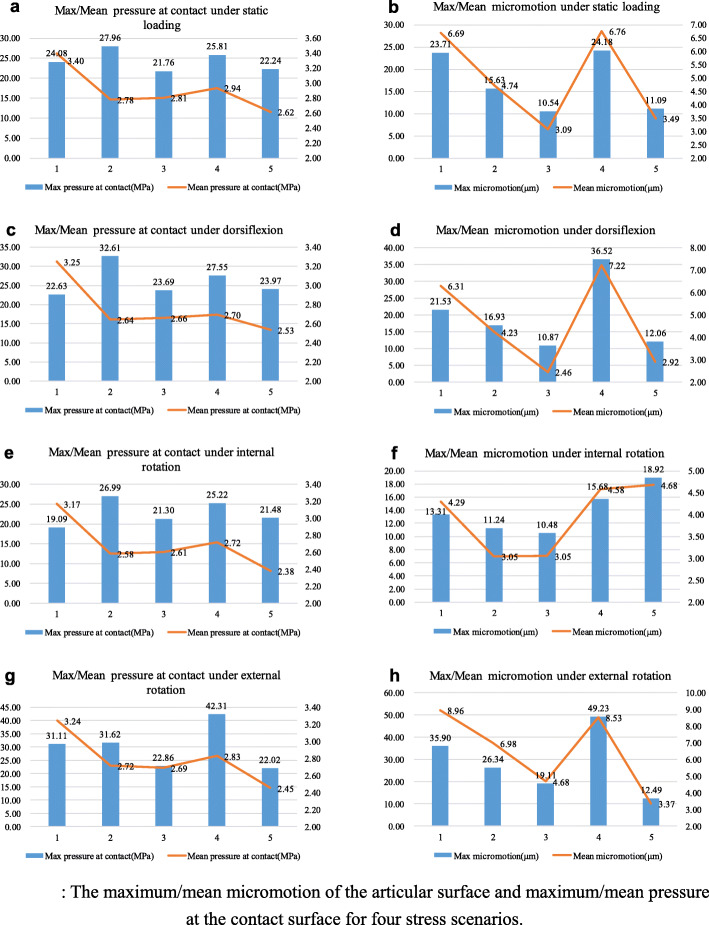
The maximum/mean micromotion of the articular surface and maximum/mean pressure at the contact surface for four stress scenarios

For static loading and dorsiflexion, layouts 3 and 5 had the lowest and second lowest maximum and mean micromotion on the articular surface, and layout 4 had the highest. Under internal torsion, layouts 3 and 2 had the lowest and second lowest maximum and mean micromotion on the articular surface, while layout 5 had the highest. In addition, layouts 5 and 3 with external torsion exhibited the lowest and second lowest maximum and mean micromotion, respectively, and layout 4 exhibited the highest (Fig. 3). The maximum and mean micromotions of the screws displayed the similar pattern, with layouts 3 and 5 having lower micromotion under static loading, dorsiflexion, and external rotation and layouts 3 and 2 under internal rotation (see Additional file 1).

**Fig. 4:**
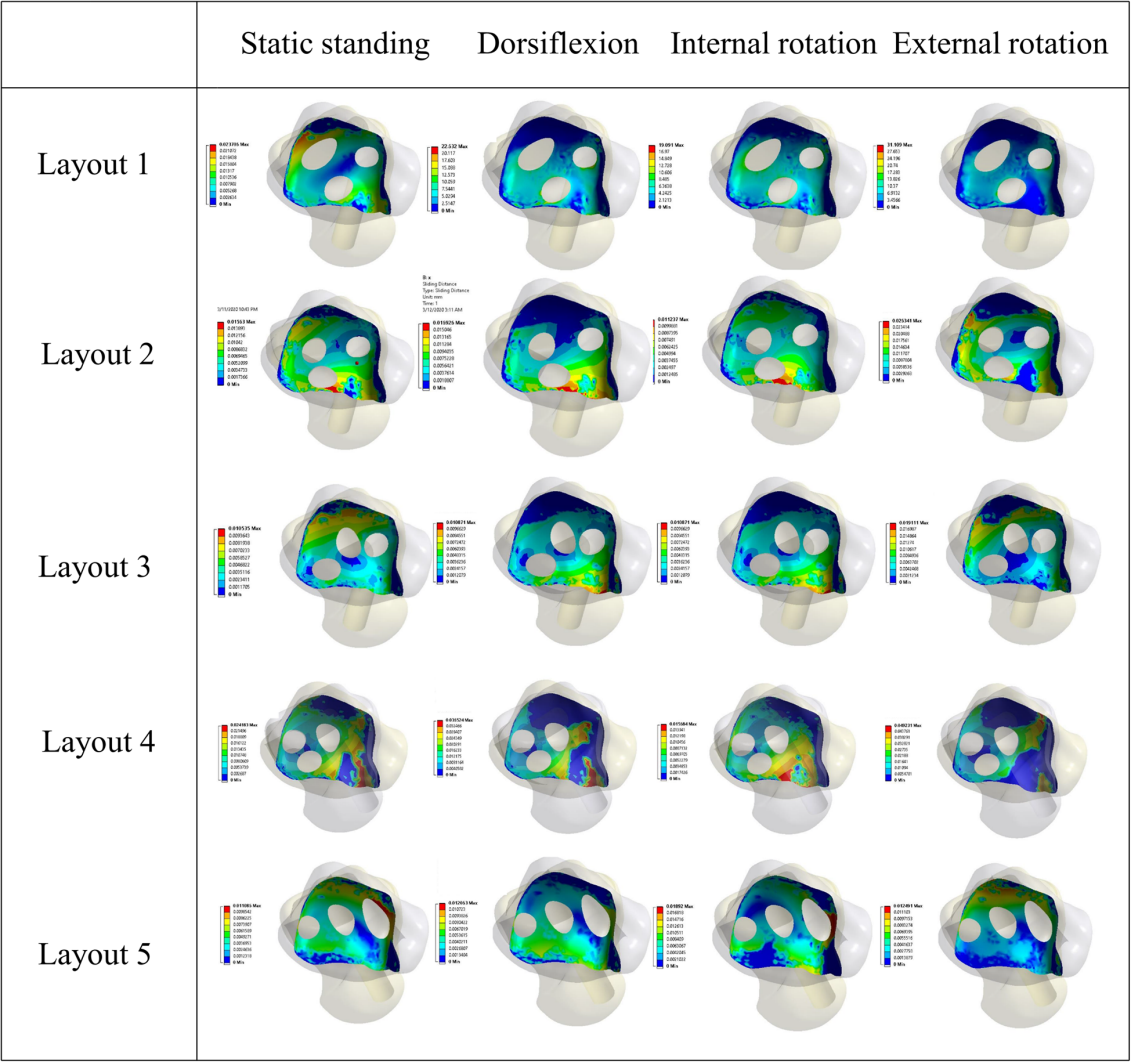
Micromotion contour plot (bottom view) showing the distribution of the micromotions on the contact surface of the tibia

Overall, the maximum and mean micromotions on the articular surface for layouts with the posteromedial home-run screw were 17.96 μm (SD 7.49 μm) and 4.88 μm (SD 1.89 μm), respectively. The maximum and mean micromotions on the articular surface for configurations with the posterolateral home-run screw were 22.52 μm (SD 12.8 μm) and 5.19 μm (SD 1.92 μm), respectively. The micromotion contour plot showed that layouts 1–3 had better screw distributions on the articular surface, as the three screw penetration points were all located in the anterolateral portion of the articular surface for layout 4 and were almost in rectilinear form rather than triangular arrangement for layout 5 (Fig. 4).

With regard to the pressure at the contact surface, layouts 3 and 5 showed better biomechanical performance than others for all loading conditions (Fig. 3), and this is consistent with the overall maximum and mean von Mises stress values at the tibia and the talus overall (see Additional file 2).

## Discussion

AAA is an effective and minimally invasive method for the treatment of advanced traumatic ankle arthritis, during which three cannulated compression screws are percutaneously inserted to fuse the tibiotalar joint. Sometimes three screws might block each other due to the small area of the tibiotalar joint surface. However, there are few articles illustrating how to insert three screws without the screws disturbing each other. We developed five possible configurations of tripod fixation in arthroscopic ankle arthrodesis that avoided the collision of screws and compared biomechanical stability through the finite element method. We found configurations with the posteromedial home-run screw presented lower total displacement (especially configuration 3) and better screw distributions on the articular surface than those with the posterolateral home-run screw.

In recent years, total ankle arthroplasty (TAA) has challenged the status of ankle arthrodesis with the promotion of third-generation prostheses [17, 18]. TAA can maintain a normal range of motion of the ankle joint, prevent degeneration of adjacent joints, and help patients restore normal gait and function [19, 20]. However, ankle replacement is not as mature as hip and knee replacement due to factors including prosthetic design and anatomical characteristics [21, 22]. A recent meta-analysis compared ankle arthrodesis with ankle replacement using third-generation prostheses and suggested that the overall complication rate was higher in the AAA group, but the revision rate was higher in the TAA group [19]. Therefore, the authors believe that the treatment options for advanced ankle osteoarthritis should be determined based on specific circumstances.

A home-run screw refers to a screw that originates from the posterior tibia and penetrates the talar head-neck junction and yields the best possible anchoring force. As advanced ankle osteoarthritis is often accompanied by anterior dislocation of the talus, a home-run screw can ensure that the talus is maximally held and maintained in the normal position [12]. Some authors argue that the posterolateral home-run screw is more prone to cause screw collision because it is positioned more laterally [12]. From a geometric perspective, the posteromedial home-run screw passes the medial portion of the talar articular surface, leaving more space for the other two screws to be inserted, while the posterolateral home-run screw runs anteromedially from the posterolateral aspect of the tibia and passes the middle part of the talar articular surface, making it more difficult to insert other screws. Meanwhile, a cadaver study by de Cesar Netto et al. [23] suggested a 73% risk of injury to the sural nerve with a percutaneous posterolateral home-run screw. In contrast, the insertion of the posteromedial home-run screw also risks damaging structures in the malleolar canal, but the risk can be minimized by cautious dissection and protection of the posterior tibial nerve and vessels because the entry point is relatively superficial. In addition, screw configurations with the posteromedial home-run screw had lower total displacement (especially configuration 3) and better screw distributions on the articular surface than those with the posterolateral home-run screw according to this study, which indicated that the posteromedial home-run screw may provide better biomechanical performance.

Layouts 3 and 5 had a relatively low maximum and mean micromotion on the articular surface for all loading conditions, except that layout 5 under internal torque exhibited the highest maximum and mean micromotion. This may be due to the relative position of the screws. The posterolateral home-run screw is driven into the talus head from the posterolateral aspect of the distal tibia. The screw trajectory is expected to be longer than that of the posteromedial home-run screw and consequently requires more space, making it difficult to implant the remaining two screws or to form a triangular relationship; thus, anti-rotation stability is impaired.

The present study has some limitations. First, the findings of this study were based on the engineering design software and a finite element model through the 3D reconstruction of CT scan images of a healthy adult and were not verified by cadaveric or clinical research. Second, due to individual differences, the results of this study may not be applicable to everyone regarding the establishment of a finite element model and can only be used for reference. In addition, the three cancellous bone screws were simplified into cylinders without threads, and cartilage, ligaments, muscles, and other tissues were not taken into account in this model. The effects of actual screw geometry on the results should be further investigated, and future expansions of the model should add more anatomical structures for an accurate simulation of the human ankle. Finally, the five screw configurations do not fully represent all the possibilities of three-screw fixation. Any modification in the three-dimensional geometry may change the final stress distribution analysis so that the recommended layout 3 may no longer be the optimal global solution; therefore, further research is still needed.

Despite the limitations, the results of this study showed that there were five possible screw configurations of tripod fixation in arthroscopic ankle arthrodesis, and configurations with the posteromedial home-run screw (layouts 1–3) were not prone to cause screw collision and were more biomechanically stable, especially layout 3. The entry point of the posteromedial home-run screw is relatively superficial, and the insertion process is not difficult. However, further cadaveric and clinical studies are needed to verify these findings in the future.

## Conclusion

Screw configurations with the posteromedial home-run screw avoid collision and are more stable biomechanically than those with the posterolateral home-run screw. Thus, inserting the home-run screw through the posteromedial approach is recommended for clinical practice.

## Supplementary information

**Additional file 1.** The maximum/mean screw micromotion for four stress scenarios.

**Additional file 2.** The maximum/mean von Mises stress values at the tibia and the talus for four stress scenarios.

## Data Availability

All data and materials were in full compliance with the journal’s policy.

## Abbreviations

3D: Three-dimensional
AAA: Arthroscopic ankle arthrodesis
CT: Computed tomography
TAA: Total ankle arthroplasty

## References

[1] Ahmad J, Raikin SM (2008). Ankle arthrodesis: the simple and the complex. Foot Ankle Clin.

[2] Elmlund AO, Winson IG (2015). Arthroscopic ankle arthrodesis. Foot Ankle Clin.

[3] Quayle J, Shafafy R, Khan MA, Ghosh K, Sakellariou A, Gougoulias N (2018). Arthroscopic versus open ankle arthrodesis. Foot Ankle Surg.

[4] Vaishya R, Azizi AT, Agarwal AK, Vijay V (2017). Arthroscopic assisted ankle arthrodesis: a retrospective study of 32 cases. J Clin Orthop Trauma.

[5] Park JH, Kim HJ, Suh DH, Lee JW, Kim HJ, Oh MJ, Choi GW (2018). Arthroscopic versus open ankle arthrodesis: a systematic review. Arthroscopy.

[6] Kamijo S, Kumai T, Tanaka S, Mano T, Tanaka Y (2017). Comparison of compressive forces caused by various cannulated cancellous screws used in arthroscopic ankle arthrodesis. J Orthop Surg Res.

[7] de Leeuw PA, Hendrickx RP, van Dijk CN, Stufkens SS, Kerkhoffs GM (2016). Midterm results of posterior arthroscopic ankle fusion. Knee Surg Sports Traumatol Arthrosc.

[8] Somberg AM, Whiteside WK, Nilssen E, Murawski D, Liu W (2016). Biomechanical evaluation of a second generation headless compression screw for ankle arthrodesis in a cadaver model. Foot Ankle Surg.

[9] Alonso-Vazquez A, Lauge-Pedersen H, Lidgren L, Taylor M (2004). Initial stability of ankle arthrodesis with three-screw fixation. A finite element analysis. Clin Biomech (Bristol, Avon).

[10] Goetzmann T, Mole D, Jullion S, Roche O, Sirveaux F, Jacquot A (2016). Influence of fixation with two vs. three screws on union of arthroscopic tibio-talar arthrodesis: comparative radiographic study of 111 cases. Orthop Traumatol Surg Res.

[11] Duan X, Yang L, Yin L (2016). Arthroscopic arthrodesis for ankle arthritis without bone graft. J Orthop Surg Res.

[12] Schuberth JM, Ruch JA, Hansen ST (2009). The tripod fixation technique for ankle arthrodesis. J Foot Ankle Surg.

[13] Vazquez AA, Lauge-Pedersen H, Lidgren L, Taylor M (2003). Finite element analysis of the initial stability of ankle arthrodesis with internal fixation: flat cut versus intact joint contours. Clin Biomech (Bristol, Avon).

[14] Zhu M, Yuan CS, Jin ZM, Wang YJ, Shi YX, Yang ZJ, Tang K (2018). Initial stability and stress distribution of ankle arthroscopic arthrodesis with three kinds of 2-screw configuration fixation: a finite element analysis. J Orthop Surg Res.

[15] Zhang Y, Xu J, Wang X, Huang J, Zhang C, Chen L, Wang C, Ma X (2013). An in vivo study of hindfoot 3D kinetics in stage II posterior tibial tendon dysfunction (PTTD) flatfoot based on weight-bearing CT scan. Bone Joint Res.

[16] Keaveny TM, Hayes WC (1993). A 20-year perspective on the mechanical properties of trabecular bone. J Biomech Eng.

[17] Gross CE, Lampley A, Green CL, DeOrio JK, Easley M, Adams S, Nunley JA (2016). The effect of obesity on functional outcomes and complications in total ankle arthroplasty. Foot Ankle Int.

[18] Jung HG, Shin MH, Lee SH, Eom JS, Lee DO (2015). Comparison of the outcomes between two 3-component total ankle implants. Foot Ankle Int.

[19] Lawton CD, Butler BA, Dekker RG, Prescott A, Kadakia AR (2017). Total ankle arthroplasty versus ankle arthrodesis-a comparison of outcomes over the last decade. J Orthop Surg Res.

[20] Marks RM (2019). Mid-term prospective clinical and radiographic outcomes of a modern fixed-bearing total ankle arthroplasty. J Foot Ankle Surg.

[21] Lee GW, Lee KB (2019). Outcomes of total ankle arthroplasty in ankles with >20° of coronal plane deformity. J Bone Joint Surg Am.

[22] McKearney DA, Stender CJ, Cook BK, Moore ES, Gunnell LM, Monier BC, Sangeorzan BJ, Ledoux WR (2019). Altered range of motion and plantar pressure in anterior and posterior malaligned total ankle arthroplasty: a cadaveric gait study. J Bone Joint Surg Am.

[23] de Cesar NC, Roberts L, Staggers J, Smith W, Lee S, Dos Santos AG, Pinto M, Araoye I, Hudson P, Shah A (2018). Ankle fusion percutaneous home run screw fixation:technical aspects and soft tissue structures at risk. Foot & Ankle Orthopaedics.

