# Supplementary material for: Finite element analysis of the initial stability of arthroscopic ankle arthrodesis with three-screw fixation: posteromedial versus posterolateral home-run screw
Source: J Orthop Surg Res. 2020 Jul 10;15:252. doi: 10.1186/s13018-020-01767-7 (PMC7350182; doi:10.1186/s13018-020-01767-7)
Supplement: Supplementary file 2 — Additional file 2. The maximum/mean von Mises stress values at the tibia and the talus for four stress scenarios. [file 13018_2020_1767_MOESM2_ESM.doc]

Additional file 2: The maximum/mean von Mises stress values at the tibia and the talus for four stress scenarios.
